# Neural Correlates of Mirror Visual Feedback-Induced Performance Improvements: A Resting-State fMRI Study

**DOI:** 10.3389/fnhum.2017.00054

**Published:** 2017-02-06

**Authors:** Viola Rjosk, Jöran Lepsien, Elisabeth Kaminski, Maike Hoff, Bernhard Sehm, Christopher J. Steele, Arno Villringer, Patrick Ragert

**Affiliations:** ^1^Department of Neurology, Max Planck Institute for Human Cognitive and Brain SciencesLeipzig, Germany; ^2^Cerebral Imaging Centre, Department of Psychiatry, Douglas Mental Health Institute, McGill UniversityMontreal, QC, Canada; ^3^Mind and Brain Institute, Charité and Humboldt UniversityBerlin, Germany; ^4^Institute for General Kinesiology and Exercise Science, University of LeipzigLeipzig, Germany

**Keywords:** mirror visual feedback (MVF), resting state functional connectivity, motor performance, neurorehabilitation, neuroplasticity

## Abstract

Mirror visual feedback (MVF) is a promising approach to enhance motor performance without training in healthy adults as well as in patients with focal brain lesions. There is preliminary evidence that a functional modulation within and between primary motor cortices as assessed with transcranial magnetic stimulation (TMS) might be one candidate mechanism mediating the observed behavioral effects. Recently, studies using task-based functional magnetic resonance imaging (fMRI) have indicated that MVF-induced functional changes might not be restricted to the primary motor cortex (M1) but also include higher order regions responsible for perceptual-motor coordination and visual attention. However, aside from these instantaneous task-induced brain changes, little is known about learning-related neuroplasticity induced by MVF. Thus, in the present study, we assessed MVF-induced functional network plasticity with resting-state fMRI (rs-fMRI). We performed rs-fMRI of 35 right-handed, healthy adults before and after performing a complex ball-rotation task. The primary outcome measure was the performance improvement of the untrained left hand (LH) before and after right hand (RH) training with MVF (mirror group [MG], *n* = 17) or without MVF (control group [CG], *n* = 18). Behaviorally, the MG showed superior performance improvements of the untrained LH. In resting-state functional connectivity (rs-FC), an interaction analysis between groups showed changes in left visual cortex (V1, V2) revealing an increase of centrality in the MG. Within group comparisons showed further functional alterations in bilateral primary sensorimotor cortex (SM1), left V4 and left anterior intraparietal sulcus (aIP) in the MG, only. Importantly, a correlation analysis revealed a linear positive relationship between MVF-induced improvements of the untrained LH and functional alterations in left SM1. Our results suggest that MVF-induced performance improvements are associated with functional learning-related brain plasticity and have identified additional target regions for non-invasive brain stimulation techniques, a finding of potential interest for neurorehabilitation.

## Introduction

### Behavioral Findings of Mirror Visual Feedback

Motor recovery after stroke depends on the intrinsic properties of the central nervous system to reorganize its structure, function and connections. Therefore, it is important that rehabilitation programs facilitate neural plasticity by including repetitive, intensive and task-relevant movement training (Takeuchi and Izumi, 2013). Mirror visual feedback (MVF) has been successfully applied to enhance motor performance without training not only in healthy adults (Nojima et al., 2012; Hoff et al., 2015; von Rein et al., 2015) but also in patients suffering from focal brain lesions (Altschuler et al., 1999; Yavuzer et al., 2008; Dohle et al., 2009; Michielsen et al., 2011; Thieme et al., 2012). For MVF-training, participants perform a task while observing the movements of their active limb in an orthogonally placed mirror providing MVF. Interestingly, MVF-induced performance improvements do not seem to be affected by hand dominance (Rjosk et al., 2015) which indicates that both hands can be equally well used for MVF-training.

However, it is known that to some extent, unilateral skill training results in performance gains of both the trained and untrained limb (Obayashi, 2004; Perez et al., 2007; Kwon et al., 2013). In the literature, this phenomenon called intermanual transfer, has been described for multiple motor tasks, like strength training (Carroll et al., 2006), sequential pinch force tasks (Camus et al., 2009) and reaching movements (Criscimagna-Hemminger et al., 2003). It seems to be influenced by several factors, like structural integrity of the corpus callosum or complexity of the task (Bonzano et al., 2011). Interestingly, neural mechanisms underlying intermanual transfer seem to be divergent from those mediating MVF-induced performance improvements (Nojima et al., 2012, 2013) and further studies are needed to distinguish these mechanisms/phenomena.

### Findings of Studies Combining Non-Invasive Brain Stimulation and Mirror Visual Feedback

It has been shown that MVF is associated with functional alterations in primary motor cortex (M1) representing the resting hand as assessed with transcranial magnetic stimulation (TMS; Nojima et al., 2012; Kumru et al., 2016). Furthermore, studies applying facilitatory transcranial direct current stimulation (a-tDCS) over M1 representing the resting hand showed enhanced mirror illusion as well as superior MVF-induced performance improvements in healthy participants relative to sham stimulation (Hoff et al., 2015; Jax et al., 2015; von Rein et al., 2015).

### Neural Correlates of Mirror Visual Feedback as Assessed with fMRI

Analysis of tasked-based functional magnetic resonance imaging (fMRI) providing MVF during a grasping task or finger-thumb opposition task showed that functional alterations are not limited to M1 but also involve other motor-related brain areas such as the premotor cortex (PMC) and the supplementary motor area as well as the primary somatosensory cortex (S1; Hamzei et al., 2012; Fritzsch et al., 2014). Moreover, also higher order areas of perceptual-motor coordination and visual attention seem to be involved in the processing of MVF such as the superior temporal gyrus and the secondary visual cortex (V2) ipsilateral to the moving hand as well as the bilateral anterior intraparietal sulcus (aIP; Matthys et al., 2009; Numata et al., 2013).

However, in these previous studies, brain activation was captured during MVF performance using task-based fMRI, thus one can only draw conclusions on instantaneous brain adaptations. Resting-state fMRI (rs-fMRI), however, provides unique insights in functional plasticity beyond instantaneous task-induced brain changes. Resting-state functional connectivity (rs-FC) represents neuronal activity that is not attributable to specific task-evoked fluctuations in blood-oxygen-level-dependent (BOLD) signal but represents signals that are intrinsically generated by the brain (Biswal et al., 1995; Fox and Raichle, 2007). These low-frequency changes are not random but highly organized, temporally correlated across distinct brain regions and of behavioral relevance (Buckner and Vincent, 2007; Guerra-Carrillo et al., 2014). Furthermore, it has been shown that these activity patterns reflect the subsequent processing and consolidation of information gained from earlier learning (Miall and Robertson, 2006; Peigneux et al., 2006). Albert et al. (2009) demonstrated that after a visuomotor perturbation task, subsequent resting activity in task-specific networks was modulated. Interestingly, changes in rs-FC in this study reflected learning or adaptation processes rather than activity changes induced by movement execution as pure motor performance did not elicit any changes in rs-FC. Hence, we used rs-fMRI to describe learning-induced neuroplasticity (Buckner and Vincent, 2007; Guerra-Carrillo et al., 2014), without confound of pure motor performance, because participants perform no task during scanning (Albert et al., 2009; Ma et al., 2011; Vahdat et al., 2011). Furthermore, it has been shown that resting-state networks are consistent across experimental sessions and participants (Damoiseaux et al., 2006; Shehzad et al., 2009).

Thus, the present study aims at evaluating learning-related changes in functional network connectivity induced by MVF and aims at identifying brain regions mediating MVF-induced performance improvements of the untrained hand. We hypothesized that MVF (mirror group, MG) during right hand (RH) training results in superior performance improvement of the untrained left hand (LH) as compared to no MVF (control group, CG; Nojima et al., 2012; Hoff et al., 2015; von Rein et al., 2015). Furthermore, we hypothesized that MVF during training will result in additional rs-FC changes in MG as compared to CG. Based on previous findings, we expected significant alterations in sensorimotor as well as higher order visual areas (Matthys et al., 2009; Hamzei et al., 2012; Numata et al., 2013). Additionally, we investigated whether these rs-FC changes are associated with the individual behavioral gains of the untrained LH.

## Materials and Methods

### Participants

Thirty-five healthy and task naïve participants (mean age: 26.91 ± 0.61 years; 16 females) took part in the study. All participants were right-handed as assessed with the Edinburgh Handedness Inventory (mean handedness score of 87.91 ± 2.79; Oldfield, 1971). The study was performed in accordance with the Declaration of Helsinki and was approved by the local ethics committee of the University of Leipzig. All participants gave their written informed consent. None of the participants was taking any centrally acting medication and none of the participants had a history of neurological illness. Highly skilled musicians and sportsmen were excluded from the study as well as participants who had contraindications for MRI measurements. Total hours of sports per week and hours of fine-motor training per week were assessed with a questionnaire. Seventeen participants were randomly assigned to the MG, the group that performed the ball-rotation task with MVF. The other 18 participants were assigned to the CG and performed the same task without MVF (see also Table 1 for group demographics). Before and after the experiment, all participants rated their levels of attention, fatigue and discomfort on a visual analog scale (VAS).

**Table 1:** **Group demographics**.

	Age (years)	Gender (female/male)	LQ	Sports/week (hours)	Fine-motor/week (hours)
**MG** *n* = 17	26.53 ± 0.95	8/9	87.82 ± 3.92	2.71 ± 0.47	0.18 ± 0.18
**CG** *n* = 18	27.28 ± 0.75	8/10	88.00 ± 4.08	3.44 ± 0.75	0.33 ± 0.28

*Hours of sports per week and hours of fine-motor training per week were assessed with a questionnaire, handedness (Laterality Quotient, LQ) was assessed with the Edinburgh Handedness Scale (range: −100 (full left-handed) to +100 (full right-handed)). Statistical analysis revealed no significant differences in age, gender, LQ, sports/week or fine-motor training/week between groups. All values are depicted as mean ± standard error of the mean. MG, mirror group; CG, control group*.

### Experimental Procedures

#### Study Design

**Figure 1:**
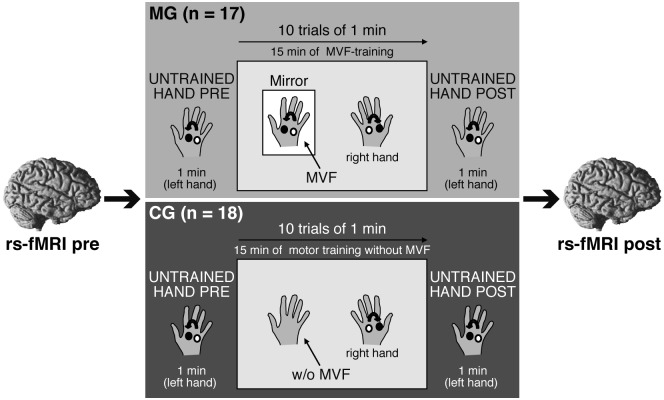
**Experimental setup and design.** Participants participated in one experimental session. A resting-state functional magnetic resonance imaging (rs-fMRI) was acquired before (rs-fMRI_pre) and after (rs-fMRI_post) a complex ball-rotation task which was performed with two cork balls outside of the scanner. Here, participants in both groups first rotated the balls with their left hand (LH) in a counterclockwise direction for 1 min (LH_pre), followed by a 15 min training phase with the right hand (RH) in a clockwise direction (10 trials of 1 min each with 30 s breaks in between). The performing RH was covered to prevent direct view. After the training period, the performance of the LH was retested (LH_post). Only the condition used in the training phase of the ball-rotation task differed between groups: participants in the mirror group (MG) received mirror visual feedback (MVF) during the training period of the RH, whereas participants in the control group (CG) watched their resting LH. See text for details.

The study consisted of one experimental session per subject. Here, we first acquired a rs-fMRI (rs-fMRI_pre) and a T1-weighted anatomical image of each participant. Then, MG and CG performed a complex ball-rotation task outside of the scanner immediately followed by another rs-state fMRI (rs-fMRI_post) ~10 min after termination of the ball-rotation task. The only difference between groups was the condition used in the ball-rotation task: participants in MG received MVF during RH training, whereas participants in CG performed the same training without MVF (Figure 1). A between-group design was chosen to ensure that all subjects were task naïve before participating in the ball-rotation task. See below for a detailed description of the complex ball-rotation task.

#### Ball-Rotation Task and MVF

We adapted the ball-rotation task introduced by Nojima et al. (2012). In short, participants were seated at a desk with their arms extended in front of them and their hands in a relaxed pronated position. To assess baseline performance, participants in both groups were asked to rotate two cork balls (diameter 30 mm; weight 10 g) for 1 min with their LH in a counterclockwise orientation as quickly as possible (LH_pre) while observing the performance of the LH. Subsequently, the training period was conducted: participants were instructed to rotate the balls with their RH but in a clockwise orientation as quickly as possible. Here, however, a wooden barrier placed over the RH prevented a direct view of the RH. Participants in MG were asked to observe the movements of their RH in a mirror (providing MVF) placed between their arms. The MVF-training was performed for 10 trials of 1 min each, separated by 30 s breaks. This led to a total of 15 min of MVF-training. During MVF-training, participants were instructed to concentrate on the mirror image and to relax their LH behind the mirror. Participants in CG performed the same training of the RH (10 trials of clockwise rotation, trial length 1 min with 30 s breaks), except that there was no mirror present. Participants in CG were instructed to observe their resting LH while a direct view of the RH during training phase was prevented by a wooden barrier placed over the RH. After this training phase, performance of the untrained LH was retested in both groups in the same manner as for LH_pre by rotating the balls with the LH in a counterclockwise direction for 1 min (LH_post). Motor performance was videotaped throughout the experiment. The number of ball-rotations/min was counted by an experimenter, who was blinded to the study procedures, and analyzed offline to assess motor dexterity.

#### MRI Data Acquisition

We used a Siemens Magnetom Tim Trio 3 T scanner equipped with a 32-channel head coil. Each experimental session consisted of two scans of echo-planar-imaging (EPI): one scan before the ball-rotation task (rs-fMRI_pre; ~7.6 min) and one scan after (rs-fMRI_post; ~7.6 min). Each scan was acquired with a total of 200 whole-brain volumes using the following parameters: acquisition matrix 64 × 64, 3 mm isotropic voxel, 1 mm gap between slices, 34 slices, TR = 2300 ms. For co-registration, we acquired T1-weighted anatomical images (MP2RAGE; ~5.12 min) before the ball-rotation task with the following parameters: voxel size = 1 mm × 1 mm × 1 mm, 176 sagittal slices, FOV = 256 × 240 mm. Participants were instructed to relax but to stay awake while keeping their eyes closed during image acquisition.

#### MRI Data Analysis

Analysis of the rs-fMRI data was performed with the FMRIB Software Library (FSL 5.0^1^), utilizing the independent component analysis (ICA)-AROMA pipeline, the fastECM toolbox (Wink et al., 2012) and SPM12^2^ running in MATLAB version 8.6^3^. First, the T1-weighted images were segmented using SPM12, and a skull-stripped version was created with fslmaths. Second, standard motion correction, spatial smoothing with a Gaussian kernel of 6 mm FWHM and linear registration of functional and T1-weighted images to each other and to the MNI space were performed. To further detect and remove motion-related artifacts ICA-AROMA (Pruim et al., 2015a,b) was used to perform an ICA on the functional data to identify and remove head motion related components by employing predefined temporal (high frequency content and maximum correlation) as well as spatial features (edge and cerebrospinal fluid fraction). Subsequently, additional nuisance correction was performed by regressing out signal from white matter and cerebrospinal fluid (physiological noise; Fox and Raichle, 2007) and by high pass filtering (0.01 Hz). Within the preprocessing, the functional data was also resampled at a resolution of 2 × 2 × 2 mm^3^ as this is the standard in most fMRI analyses.

For the analysis of functional connectivity, the eigenvector centrality maps (ECM) approach was used. Eigenvector centrality can quantify the relative importance, or centrality, of an individual node on a network as a whole. The centrality is high if a node is connected to many nodes that themselves are “central” (Lohmann et al., 2010). Hereby, the importance of points in brain networks can be measured and visualized (Lohmann et al., 2010). Centrality analyses are supposed to enable the interpretability of connectivity matrices used in graph analyses combined with the high spatial resolution of voxel-based methods (Wink et al., 2012). ECM, in particular, were used for our data analysis for its exploratory whole brain approach independently from predefined seed regions (Lohmann et al., 2010). Using the preprocessed functional data, an ECM analyses was performed for each subject and each scan time point (rs-fMRI_pre and rs-fMRI_post) separately using the fastECM toolbox (Wink et al., 2012). The fastECM algorithm was chosen for its shorter computation times and lower storage requirements for high-resolution fMRI data. Within the preprocessed functional images, the voxel-wise connectivities between all pairs of voxels were computed with the fastECM program within a study-specific gray matter mask. To create this study-specific mask, the individual gray matter masks derived from segmenting the T1-weighted images were added up. Then a threshold was applied to include only voxels that contained gray matter from all participants (total of 202519 voxel). Hence, a 3D voxel-wise ECM per subject and scan time point was created and used for further statistical analyses.

All statistical analyses were performed using SPM12. To assess the effect of MVF on the rs-FC single-subject ECM images were compared using a 2 × 2 flexible factorial design with the factors TIME (rs-fMRI_pre vs. rs-fMRI_post) and GROUP (MG vs. CG), and complemented with subsidiary *t*-tests. To analyze a potential relationship between changes in functional connectivity and behavioral improvements, a correlation analysis was performed with SPM12. First, ECM difference images (rs-fMRI_post minus rs-fMRI_pre) were created on the single subject level using the ImCalc-toolbox in SPM12. We then performed a correlation analysis per group correlating the difference images with the performance improvement of the untrained LH.

All stated findings are significant at *p* < 0.05 with cluster-wise (*p* = 0.001) FWE correction for multiple comparisons.

### Statistical Analyses

#### Data Analyses: Ball-Rotation Task

We used the Statistical Software Package for Social Sciences (IBM SPSS Version 22) for statistical analyses of the behavioral data. The number of ball-rotations/min both for the LH_pre and LH_post as well as for the RH (trials T1–T10) during the training period was used to assess motor performance. To test for differences in baseline performance, an independent samples *t*-test was used to compare the number of ball-rotations/min of the LH_pre between groups (MG vs. CG). Subsequently, a repeated-measures ANOVA (ANOVA-RM) with factor TRIAL (LH_pre vs. LH_post) and GROUP (MG vs. CG) was performed to assess the effect of MVF during the training period on performance improvements of the untrained LH. Here, the factor TRIAL was the independent variable or the within-subject factor with two levels (LH_pre vs. LH_post). The factor GROUP was the between-subjects factor (MG vs. CG). Supporting this, independent samples *t*-tests were conducted to compare the absolute and relative amount of performance improvement of the untrained LH after the training period across groups (MG vs. CG). Paired *t*-tests, comparing LH_pre vs. LH_post, were used to evaluate the performance improvement of the untrained hand within each group. Finally, the RH performance over the whole training period was evaluated using another ANOVA-RM with factor TRIAL (T1–T10) and GROUP (MG vs. CG) to test whether participants in both groups improved their RH during training period. Again, factor TRIAL was the within-subject factor (10 levels T1–T10) and factor GROUP was the between-subjects factor (MG vs. CG). Further within- and between-group comparisons were performed using *post hoc t-tests*. A Bonferroni corrected *p-value* of <0.05 was considered to be significant. Greenhouse-Geisser correction was applied, if applicable. Behavioral data are presented as mean ± standard error (SE). The Eta-squared (*η*^2^) is reported for each ANOVA as a measure of the effect size. We considered an *η*^2^ of ≥0.02 as small, ≥0.13 medium and ≥0.26 large effect as proposed by Miles and Shevlin (2001).

## Results

Groups did not differ regarding age (*t*_(33)_ = −0.609, *p* = 0.547), gender (*t*_(33)_ = 0.151, *p* = 0.881), handedness (*t*_(33)_ = −0.031, *p* = 0.975), weekly hours of sports (*t*_(33)_ = −0.818; *p* = 0.419) or fine-motor training (*t*_(33)_ = −0.468; *p* = 0.643; see also Table 1). Prior to the experiment, participants in both groups did not differ in their levels of attention (*t*_(33)_ = −0.336, *p* = 0.739), fatigue (*t*_(33)_ = −0.097, *p* = 0.924) or discomfort (*t*_(33)_ = 0.852, *p* = 0.400).

### Behavioral Results: Ball-Rotation Task

#### Left Hand Performance

**Figure 2:**
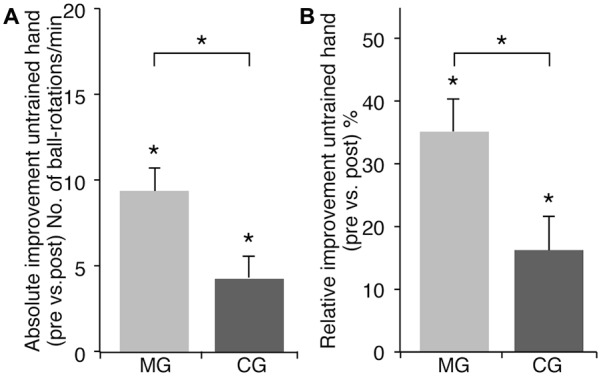
**Effect of training with or without MVF on motor performance of the untrained LH.** Note that there was no significant difference in baseline performance of the untrained LH between groups. **(A)** Absolute performance improvement of the untrained LH (ball-rotations/min). **(B)** Relative performance improvement of the untrained LH (%). Both groups improved their performance with the untrained LH significantly but there was a significantly higher gain in absolute as well as relative performance improvement in the MG as compared to the CG. The plots show mean values, and whiskers represent standard error (SE) values. *P < 0.05.

Baseline performance of the LH did not differ between MG and CG (MG: 31.82 ± 2.94; CG: 35.33 ± 3.01 ball-rotations/min, *t*_(33)_ = −0.833; *p* = 0.411). Both groups showed significant performance improvements of LH after RH training (ANOVA-RM with factor TRIAL (LH_pre vs. LH_post) × GROUP (MG vs. CG): *F*_(1, 33)_ = 7.925; *p* = 0.008; *η*^2^ = 0.194) (Figure 2). However, LH performance improvements were more pronounced in MG as compared to CG. MG improved by 9.65 ± 1.33 ball-rotations/min (*t*_(16)_ = 7.233; *p* < 0.001) (35.43 ± 5.63% [*t*_(16)_ = 6.296; *p* < 0.001]). CG improved by 4.28 ± 1.36 ball-rotations/min (*t*_(17)_ = 3.146; *p* = 0.006) (16.29 ± 5.29% [*t*_(17)_ = 3.077; *p* = 0.007]). Subsequent analyses revealed significantly higher performance improvements of the untrained hand in MG compared to CG (absolute performance improvement: *t*_(33)_ = 2.815; *p* = 0.008; relative performance improvement: *t*_(33)_ = 2.480; *p* = 0.018; Figure 2).

#### Right Hand Performance

In both groups, performing the ball-rotation task during the training phase (trials T1–T10) resulted in significant performance gains of the RH. Participants in MG improved on average by 22.94 ± 3.02 ball-rotations/min (*t*_(16)_ = −7.608; *p* < 0.001) and participants in CG by 10.17 ± 2.20 ball-rotations/min (*t*_(17)_ = −4.619; *p* < 0.001). But there was a significant difference in the absolute amount (T10 − T1) of performance improvement of the trained RH between groups (ANOVA-RM with factor TRIAL (T1–T10) × GROUP (MG vs. CG): *F*_(3.796,125.278)_ = 4.666; *p* = 0.002; *η*^2^ = 0.124). *Post hoc* between-group comparisons showed a significantly higher amount of performance improvement in MG compared to CG (*t*_(33)_ = 3.450; *p* < 0.002). However, there was no correlation between the amount of performance improvement of the untrained LH and performance improvements of the trained RH in either group (MG: *r* = 0.27; *p* = 0.299; CG: *r* = 0.213; *p* = 0.396).

For a description of behavioral data of participants in MG see also Rjosk et al. (2015).

See also Table 2 for a complete breakdown of group data of the untrained LH and trained RH in the ball-rotation task.

**Table 2:** **Group data of the untrained left hand (LH) pre and post training phase as well as of the trained right hand (RH) during training phase (trials T1–T10) in the ball-rotation task**.

	LH_pre	T1	T2	T3	T4	T5	T6	T7	T8	T9	T10	LH_post
**MG** *n* = 17	31.82 ± 2.94	23.00 ± 4.43	27.94 ± 4.47	33.00 ± 4.37	36.41 ± 3.46	36.35 ± 3.62	39.24 ± 3.23	40.71 ± 3.37	43.29 ± 3.25	43.82 ± 3.15	45.94 ± 3.14	41.47 ± 3.17
**CG** *n* = 18	35.33 ± 3.01	35.83 ± 2.37	39.06 ± 2.86	39.22 ± 3.09	40.78 ± 2.73	43.67 ± 3.28	44.33 ± 3.06	44.50 ± 3.36	45.00 ± 3.45	47.67 ± 3.49	46.00 ± 3.35	39.61 ± 2.80

*Behavioral data for the untrained LH and trained RH (ball-rotations/min). Participants in the MG received MVF during training phase (T1–T10), while participants in the CG watched their resting untrained LH during training phase. Performing the ball-rotation task during training phase (T1–T10) resulted in significant performance gains of the untrained LH as well as trained RH in both groups, while there was a significant higher amount of performance improvement in both hands in MG. For details, see text. Data are depicted as mean ± standard error of the mean*.

### Rs-fMRI Results: Centrality Changes

#### ECM

**Figure 3:**
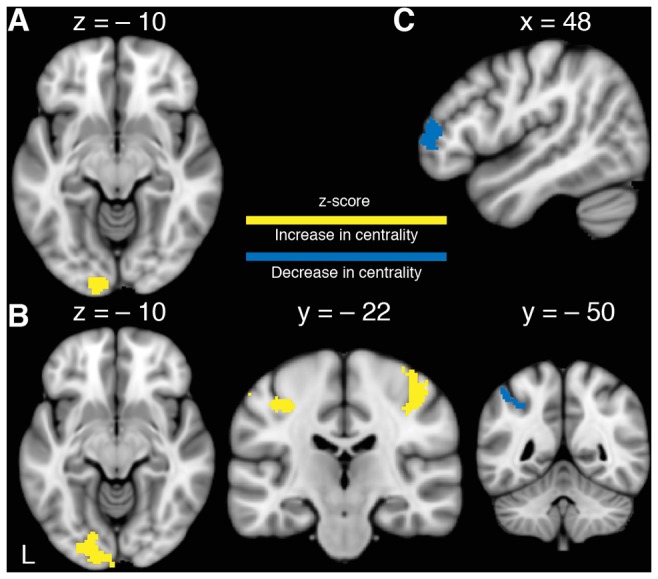
**Changes in functional connectivity. (A)** Significant TIME × GROUP interaction of changes in left visual cortex (V1, V2) revealing an increase of centrality in the MG. **(B)** Increase in centrality in MG after 15 min of training the RH with MVF in left V4 and bilateral primary sensorimotor cortex (SM1) as well as a decrease in centrality in left anterior intraparietal sulcus (aIP; Paired-*t*-tests). **(C)** Decrease in centrality in the CG in right frontopolar cortex (FPC) after RH training without MVF (Paired-*t*-test). *P*_(FWE−corr)_ < 0.05.

The interaction of TIME × GROUP showed a significant increase in centrality in MG compared to CG in left V1, V2 ipsilateral to the untrained LH (Figure 3A). There were no regions that showed a significant decrease in centrality in MG compared to CG. Subsequently, a series of *t*-tests were conducted to further investigate changes in centrality within and between groups. No significant differences in baseline rs-FC were found between groups as assessed by an independent samples *t*-test. To assess changes in centrality due to MVF, a paired-sample *t*-test for MG was conducted, which showed a significant increase in centrality in left visual areas (V4) and a non-significant trend in left PMC (*p* = 0.082) as well as a significant centrality increase in bilateral primary sensorimotor cortices (SM1). It further indicated a significant decrease in centrality in left aIP in MG (Figure 3B). The corresponding *t*-test for CG showed a decrease in centrality in the right frontopolar cortex (FPC) only (Figure 3C). This pattern of results revealed by the post vs. pre comparison of MG was mirrored in the interaction contrast TIME × GROUP by supplementary but (for multiple comparisons) non-significant changes in rs-FC in right V2, in left PMC and in bilateral SM1.

#### Correlation Analyses

**Figure 4:**
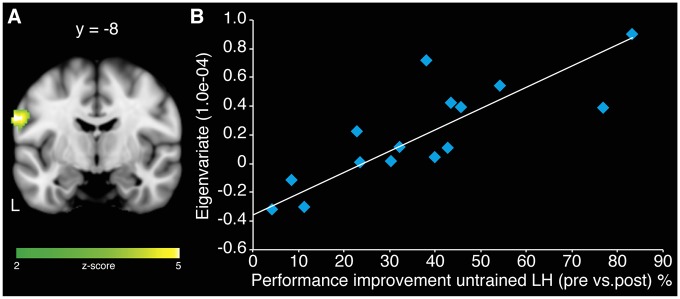
**Association of centrality changes in rs-fMRI and relative performance improvements of the untrained LH in the MG. (A)** A correlation analysis revealed a significant positive correlation between increase in centrality in left SM1 and behavioral gains of LH for MG. No such correlation could be observed for the CG. **(B)** Scatter plot diagram illustrating the correlation between the eigenvariate extracted from the peak voxel of the cluster in left SM1 and the performance improvements of the untrained LH in MG (model equation, *y* = 0.0148*x* − 0.3617; *r* = 0.842; *p* < 0.001).

A correlation analyses of centrality changes in rs-fMRI and relative performance improvements of the untrained LH revealed a significant positive relationship in left SM1 for MG (*r* = 0.842; *p* < 0.001; Figure 4). No such correlation could be observed for CG. In addition, there was no evidence for negative correlations between centrality changes and relative performance improvements within either group.

All results are summarized in Table 3.

**Table 3:** **MNI-coordinates of peak voxels of training-induced changes in eigenvector centrality (ECM)**.

Contrast	Anatomical area	MNI (*X Y Z*)			*Z*-max	No. of voxels
Interaction MG vs. CG *(TIME × GROUP)*	left V1, V2	−16	−98	−12	4.11	169
MG *(rs-fMRI_pre < rs-fMRI_post)*	left V4	−24	−84	−16	4.24	440
	left SM1	−34	−28	52	3.91	208
	right SM1	46	−20	48	3.76	221
	left PMC*	−6	−10	48	4.13	114
MG *(rs-fMRI_pre > rs-fMRI_post)*	left aIP	−42	−52	50	3.9	170
CG *(rs-fMRI_pre > rs-fMRI_post)*	right FPC	50	48	14	4.03	185
MG *(correlation analysis)*	left SM1	−66	−6	28	4.24	156

Listed are all findings that are significant at *p* < 0.05 in the corresponding contrast with cluster-wise FWE correction for multiple comparisons. *Denotes a non-significant trend only (*p* = 0.082). Please note that we found a positive correlation only in MG between left SM1 and performance improvements of the LH, for details see “Results” Section and Figure 4. MG, mirror group; CG, control group; V1–V4, visual cortices; SM1, primary sensorimotor cortices; PMC, premotor cortex; aIP, anterior intraparietal sulcus; FPC, frontopolar cortex.

## Discussion

The aim of the present study was to investigate learning-related rs-FC changes associated with MVF-induced behavioral changes in the untrained LH. In line with our hypotheses, we observed superior performance improvements in MG compared to CG. The ECM analyses revealed no differences in baseline rs-FC between groups but indicated a significant increase of centrality in left visual areas (V1, V2) in MG due to MVF-training compared to CG. Furthermore, subsidiary within group comparisons showed further functional alterations in left V4, bilateral SM1 and left aIP in MG, only.

While MG and CG both showed significant improvements in LH performance, the effect was more pronounced in MG. This finding seems to be in contrast with a control experiment performed by Nojima et al. (2012) showing no performance improvements of the untrained LH when motor training with the RH was performed without MVF in the same complex ball-rotation task. However, Reissig et al. (2015) could also not replicate the findings by Nojima et al. (2012). One potential explanation for our divergent results might be that participants in our study performed the task with the LH twice as long as participants in the study of Nojima et al. (2012) (1 min of ball-rotation instead of 30 s) giving our participants in CG more time to familiarize with the task. Furthermore, participants receiving MVF during motor training improved their dexterity above this simple familiarization effect. As we hypothesized, participants in MG showed significantly stronger behavioral gains of the untrained hand compared to CG indicating the superior effect of MVF.

Considering intermanual transfer, one might argue that MVF is not solely the driving mechanism behind the performance improvements of the untrained hand. However, the underlying neural mechanisms of these phenomena seem to be divergent. Intermanual transfer seems to be mediated via alterations in intracortical and interhemispheric inhibition (IHI) between homologous M1s (Perez et al., 2007; Camus et al., 2009), whereas Nojima et al. (2012) did not detect any alterations in IHI due to MVF. Furthermore, MVF-induced performance improvements were even shown in callosotomized patients (Nojima et al., 2013).

Contrary to our hypotheses, we did not find significant centrality changes between groups in higher order visual areas, but in left V1 and V2. However, within group comparisons revealed a significant increase in centrality in left V4 only in MG. This increase in rs-FC that we observed due to MVF-training in left visual areas is in line with previous findings of Lewisa et al. (2009), who demonstrated that visual perceptual learning can modify the resting activity between the trained visual cortex and areas involved by the task. Furthermore, regarding the underlying mechanisms for MVF in motor rehabilitation, Altschuler et al. (1999) proposed that the visual image might recruit the PMC and hereby connect the visual input to the motor system. V1, V2 and V4 are embedded into the dorsal visual stream and hereby connected to the posterior parietal cortex (PPC; Goodale and Milner, 1992). The PPC is involved in motor control and action planning and is specialized in visuomotor transformations required for visually guided movements (Fogassi and Luppino, 2005). The ability to coordinate and integrate somatosensory and visual information with motor signals is represented in areas of the inferior parietal lobule (IPL; Jeannerod and Jacob, 2005). Furthermore, the left IPL is thought to be preferentially involved in performing more complex actions as well as in storing these complex representations (Glover, 2004). Based on this, our reported activation in the left visual areas only in MG may indicate an influence of MVF on the left PPC and IPL, and thus may suggest a potential influence of MVF on storage of representations after visuomotor learning (Goodale and Milner, 1992; Glover, 2004; Fogassi and Luppino, 2005; Jeannerod and Jacob, 2005). However, results on hemispheric specific involvement of visual areas are heterogeneous as Matthys et al. (2009) found the right superior occipital gyrus (located in V2) to be associated with MVF in a fingertapping task with the RH.

Importantly, we showed a positive correlation of performance improvement of the untrained LH and centrality changes in the left SM1. This might indicate that S1 representing the trained hand plays an important role in mediating the MVF-induced effects. Interestingly, when considering the anatomical somatotopy of SM1, the cluster of the correlation analysis is not located in the hand area but in the ventral part of SM1. However, it is discussable whether the MRI resolution used in the current study is high enough to disentangle sub regions of the sensorimotor system and whether features of brain structure are an appropriate tool to assign functional properties (Wang et al., 2015).

By taking a closer look at the underlying modifications in rs-FC within each group via paired *t*-tests, we found a significant increase in centrality in MG in bilateral SM1 and left V4 and a non-significant trend towards an increase in left PMC ipsilateral to the untrained LH. Furthermore, we observed a decrease in centrality in the left aIP in MG within these analyses.

The observed rs-FC changes in MG in bilateral SM1 and the trend towards an increase in centrality in left PMC are in line with previous TMS- and task-based fMRI-studies reporting similar regions to be associated with MVF. Hamzei et al. (2012) showed MVF-specific activation changes within the bilateral PMC as well as in M1 representing the trained hand during action observation and imitation in a grasping task using task-based fMRI. Garry et al. (2005) observed enhanced excitability of the M1 representing the resting hand during MVF, and Kumru et al. (2016) found a cortical disinhibition in the ipsilateral M1 using TMS. However, Fritzsch et al. (2014) observed activity changes in S1 and argued that not M1 but S1 representing the untrained hand is directly modulated by a mirror task. In line with this, Moseley and Wiech (2009) showed an improvement of tactile discrimination in patients with complex regional pain syndrome already after one intervention with MVF, whereas improvement of motor function in patients with a hemiparesis after stroke requires repetitive training (Thieme et al., 2012). Taken these results together, we observed an increase in centrality in bilateral SM1 and a non-significant trend in the left PMC representing the trained RH.

However, it is discussable, whether these bilateral modulations in SM1 are solely due to MVF or due to bilateral sensorimotor training *per se*. Tamè et al. (2016) proposes, that S1, together with the secondary somatosensory cortex, processes tactile information from both sides of the body, especially during demanding bilateral tasks.

Considering the properties of the aIP and its connection to the ipsilateral ventral PMC enabling visually guided, object-directed hand actions (Fogassi and Luppino, 2005), our observed connectivity changes in the aIP, PMC and SM1 fit into the model of gating MVF-induced activation from the aIP into the motor system as proposed by Numata et al. (2013). They found a significant activation in the bilateral aIP in a finger-thumb opposition task-based fMRI only when MVF was provided. Contrary, we found a decrease in connectivity in left aIP. However, an anti-correlation of spontaneous BOLD activity between parts of a network (here aIP, visual and motor areas) was explained to be an efficient computational state to promote task recruitment and flexibility and may prevent the network elements from interfering with each other (Guidotti et al., 2015). Thus, our observed decrease in centrality in left aIP in MG might indicate a rise in efficiency of connectivity between involved network elements.

However, the impact of our findings within MG is limited due to the fact that functional alterations in left V4, bilateral SM1, left PMC and left aIP did not reach significance in the interaction contrast between groups.

When taking a closer look at modifications in rs-FC within CG, paired *t*-test analyses revealed only a significant decrease in centrality in the right FPC. This area is cyto-architectonically defined as the lateral part of Brodmann’s area 10 and as a component of the frontal lobe involved in decision-making (Koechlin and Summerfield, 2007). However, according to Koechlin and Hyafil (2007), the main function of FPC is “cognitive branching” meaning the ability to switch between independent tasks and to temporarily suspend a task while another is being performed. Changes in centrality in FPC may indicate that participants in CG experienced the performance of the ball-rotation task with the LH and RH as separate and independent task units. Contrarily, receiving MVF during performance of the RH training may have induced the illusion of pure LH training since participants in MG did not show changes in connectivity in FPC. However, changes in centrality in FPC also did not reach significance in the interaction contrast.

### Limitations of the Study

Since participants in our study performed the task only once and did not receive multiple MRI scans after the ball-rotation task, we cannot make inferences about the exact time course of the observed rs-FC changes. However, there is evidence that plasticity changes after training depend on the length of the training (Dayan and Cohen, 2011). Immediate changes, as assessed in the present study, might differ from later and long term changes (Taubert et al., 2010, 2011; Ma et al., 2011; Gryga et al., 2012). The fact that we showed a correlation between SM1 rs-FC changes and gains in performance of the LH does not explicitly highlight a causal relation between functional plasticity and training effects. Hence, future studies should use non-invasive brain stimulation to selectively target these brain regions (e.g., by down-regulating its activity) and thereby investigate this causal relationship in more detail.

### Conclusions and Clinical Implications

To our knowledge, the present study is the first to investigate the effect of MVF-induced performance improvements by means of rs-fMRI. In support of our hypotheses, we observed superior performance improvements in MG compared to CG. Additionally, we identified learning-related changes induced by MVF in left visual areas. Further functional alterations in left V4, left PMC and left aIP ipsilateral to the untrained LH as well as in bilateral SM1 were revealed by within group comparisons in MG, only. These findings might indicate that the effects of MVF-training are likely the result of interactions between perceptual and motor cortical regions.

Our results suggest that the hemisphere ipsilateral to the untrained hand (which would correspond to the unaffected hemisphere in patients) plays an important role in the underlying mechanism of MVF, since we found a positive correlation between centrality changes in left SM1 and performance improvements of the untrained LH. In line with this, Deconinck et al. (2015) hypothesized that MVF-induced recruitment of ipsilateral motor pathways might attribute to the behavioral gains due to MVF. Interestingly, activity in ipsilateral pathways has been discussed in the context of stroke to be beneficial for motor recovery (Benecke et al., 1991; Carr et al., 1994; Schwerin et al., 2008).

Since we showed that MVF is capable of inducing learning-related neuroplastic modifications indicated by changes in rs-FC, our results might be of clinical relevance. Our findings support the application of MVF in neurorehabilitation to facilitate neural plasticity in patients suffering from unimanual motor impairments and have identified additional target regions for non-invasive brain stimulation techniques.

## Author Contributions

VR, PR and AV designed the study. VR performed the experiment. PR, VR, CJS and JL analyzed the data. VR, EK, MH, BS, JL, CJS and PR interpreted results of the experiment; edited and revised the manuscript. PR and VR drafted the manuscript. All authors approved the final version of the manuscript.

## Conflict of Interest Statement

The authors declare that the research was conducted in the absence of any commercial or financial relationships that could be construed as a potential conflict of interest.

## Footnotes

^1^http://www.fmrib.ox.ac.uk/fsl

^2^http://www.fil.ion.ucl.ac.uk/spm/software/spm12

^3^http://mathworks.com

## Abbreviations

aIP: anterior intraparietal sulcus
CG: control group
ECM: eigenvector centrality maps
fMRI: functional magnetic resonance imaging
FPC: frontopolar cortex
IHI: interhemispheric inhibition
IPL: inferior parietal lobule
LH: left hand
M1: primary motor cortex
MG: mirror group
MVF: mirror visual feedback
PMC: premotor cortex
PPC: posterior parietal cortex
RH: right hand
Rs-FC: resting-state functional connectivity
Rs-fMRI: resting-state functional magnetic resonance imaging
S1: primary somatosensory cortex
tDCS: transcranial direct current stimulation
TMS: transcranial magnetic stimulation
V1–4: visual cortices.
